# Tuberosity refixation in reverse proximal humerus fracture arthroplasty: a prospective cohort study with two-year follow-up

**DOI:** 10.1007/s00402-026-06303-6

**Published:** 2026-06-26

**Authors:** Cornelia Hildegard Oberrauter, Florian Hess, JoEllen Welter, Hans-Christoph Pape, Saskia Mayer, Alexander Dullenkopf, Ruben Mazzucchelli, Ivan Marintschev

**Affiliations:** 1 University Department of Trauma, Hand and Reconstructive Surgery, Friedrich-Schiller-University Jena, University Hospital Jena, Jena, Germany; 2 Department of Orthopaedic Surgery and Traumatology, Cantonal Hospital Frauenfeld, Frauenfeld, Switzerland; 3 Institute for Anesthesia, Spital Thurgau Frauenfeld, Frauenfeld, Switzerland; 4 Gelenkzentrum Winterthur (GZW), Orthopädie und Unfallchirurgie, Winterthur, Switzerland; 5 Department of Traumatology, USZ, University Hospital of Zürich, Zürich, Switzerland

**Keywords:** Reverse shoulder arthroplasty, proximal humerus fracture, tuberosities, shoulder function

## Abstract

**Introduction:**

To evaluate the outcomes of a simplified tuberosity refixation technique using a two-suture construct in patients with complex proximal humerus fractures treated with reverse shoulder arthroplasty (RSA). The technique reduces the number of fixation sutures rather than the traditional four to five, aiming to decrease operative complexity while promoting greater tuberosity (GT) healing and improving the healing rate. A secondary exploratory analysis compared clinical and radiological outcomes between cemented and uncemented humeral stem fixation.

**Materials and methods:**

This prospective study included 73 patients who underwent RSA (Global Unite Fracture Reverse System, DePuy Synthes, Warsaw, Indiana, USA). Clinical and radiological outcomes were assessed at two-year follow-up. Bone quality was evaluated using the Deltoid Tuberosity Index (DTI). Functional outcomes and GT healing were analyzed, and stem fixation type was assessed in a secondary subgroup analysis.

**Results:**

The mean patient age was 79 ± 8 years (range 61–95), and 65 (89%) were female. Osteoporotic bone quality (DTI < 1.4) was observed in 51 (70%) of cases. At two years, the median outcome scores were: Subjective Shoulder Value, 90 (IQR 80–95); Constant-Murley score, 76 (IQR 73–81); active forward flexion, 140° (IQR 120–160); abduction, 140° (IQR 120–160); external rotation, 8 (IQR 8–8); and internal rotation, 6 (IQR 6–8). GT healing occurred in 68 (93%) patients. Dislocation occurred in 5 (7%) patients. Complications occurred in 4 (5.5%) patients, and 1 (1.4%) required revision surgery. In the exploratory comparison, cemented stems demonstrated slightly greater forward flexion (*p* = 0.02) and abduction (*p* = 0.045), without clinically relevant differences between fixation types.

**Conclusions:**

The simplified two-suture greater tuberosity refixation technique achieved high GT healing rates and favorable functional outcomes, supporting its use in RSA for complex proximal humerus fractures. Stem fixation method did not have a clinically relevant influence on outcomes.

## Introduction

A growing proportion of older adults has led to increasing rates of osteoporosis and associated fragility fractures [1, 2]. Among these, proximal humerus fractures represent the third most common non-vertebral fracture in the elderly, resulting in substantial morbidity and healthcare burdens [3, 4]. The incidence of these fractures is projected to triple over the next 30 years [5].

Reverse shoulder arthroplasty (RSA) has become a widely established treatment for complex proximal humerus fractures, especially in older adults with compromised bone quality or fractures at high risk of avascular necrosis [4, 6–12]. RSA enhances deltoid lever arm function by medializing the center of rotation and lowering the humerus, thereby improving forward flexion and abduction (Grammont design). In many cases, the integrity of the deltoid muscle is more critical than an intact rotator cuff in determining postoperative shoulder function [13–15].

Greater tuberosity (GT) healing plays a crucial role in achieving optimal RSA outcomes, contributing to joint stability and reducing the risk of implant-related complications. While some researchers have questioned the value of tuberosity reattachment [6, 16, 17], accumulating evidence indicates that anatomical refixation facilitates preservation of the infraspinatus and teres minor muscles, which in turn contributes to improved external rotation. Additionally, the use of cement in reverse shoulder arthroplasty remains controversial, as it may offer greater initial stability in osteoporotic bone but raises concerns regarding potential complications and its impact on greater tuberosity healing. Anatomical greater tuberosity healing has been associated with improved range of motion, better functional outcomes, higher patient satisfaction, and lower rates of complications such as infection, stem loosening, and instability [14, 18–24]. Despite numerous techniques aimed at improving greater tuberosity healing, secondary migration remains a concern.

This study evaluated the outcomes of a simplified refixation technique using the Global Unite Fracture Reverse System (DePuy Synthes, Warsaw, Indiana, USA), which had previously demonstrated promising healing rates after one year in a smaller patient cohort (*n* = 30) [13]. The technique is characterized by the use of only two strong fixation sutures, in contrast to the traditional four to five sutures commonly required for tuberosity reconstruction. This streamlined approach reduces operative complexity and may create more favorable biological conditions for tuberosity ingrowth, while also being easier to teach and assist, facilitating its adoption by less-experienced surgeons. The primary aim of the study was to evaluate clinical and patient-reported outcomes and radiologic greater tuberosity healing following this simplified technique in a larger cohort with a follow-up of two years. Since humeral stem fixation may influence primary stability and thereby affect greater tuberosity healing, a secondary analysis was performed to control for this potential confounding factor by comparing cemented versus uncemented stems.

## Materials and methods

This prospective study was conducted from June 2019 to June 2022 at two affiliated Swiss acute care hospitals that together perform approximately 3,500 orthopedic surgeries per year. The study was conducted in accordance with the Declaration of Helsinki. Consecutive patients treated with reverse shoulder arthroplasty for proximal humerus fractures during the study period were assessed for enrolment based on our study’s inclusion criteria. Written informed consent was obtained from all participants prior to inclusion. Approval for this study was granted by the Ethics Committee of Eastern Switzerland (EKOS 2019 − 00414).

We included patients with acute displaced or dislocated two-, three-, and four-part fractures according to the Neer classification [25], displaced head-split fractures, and secondarily displaced fractures following failed conservative treatment [13]. Only patients treated with the described tuberosity refixation technique (Global Unite Fracture Reverse System (DePuy Synthes, Warsaw, Indiana, USA) were eligible for inclusion. Exclusion criteria included fractures older than six weeks, prior unsuccessful open reduction and internal fixation (ORIF), failed hemiarthroplasty, any revision shoulder arthroplasty, and use of prosthetic systems other than the one described above.

Osteoporosis status was evaluated preoperatively using the Deltoid Tuberosity Index (DTI) measured from conventional anteroposterior shoulder radiographs [26]. The DTI is the ratio of the outer cortical diameter to the inner (medullary canal) diameter of the humerus at the level of the deltoid tuberosity. A DTI of less than 1.4 was considered indicative of low bone quality or osteoporosis.

### Operative technique and postoperative care

**Fig. 1:**
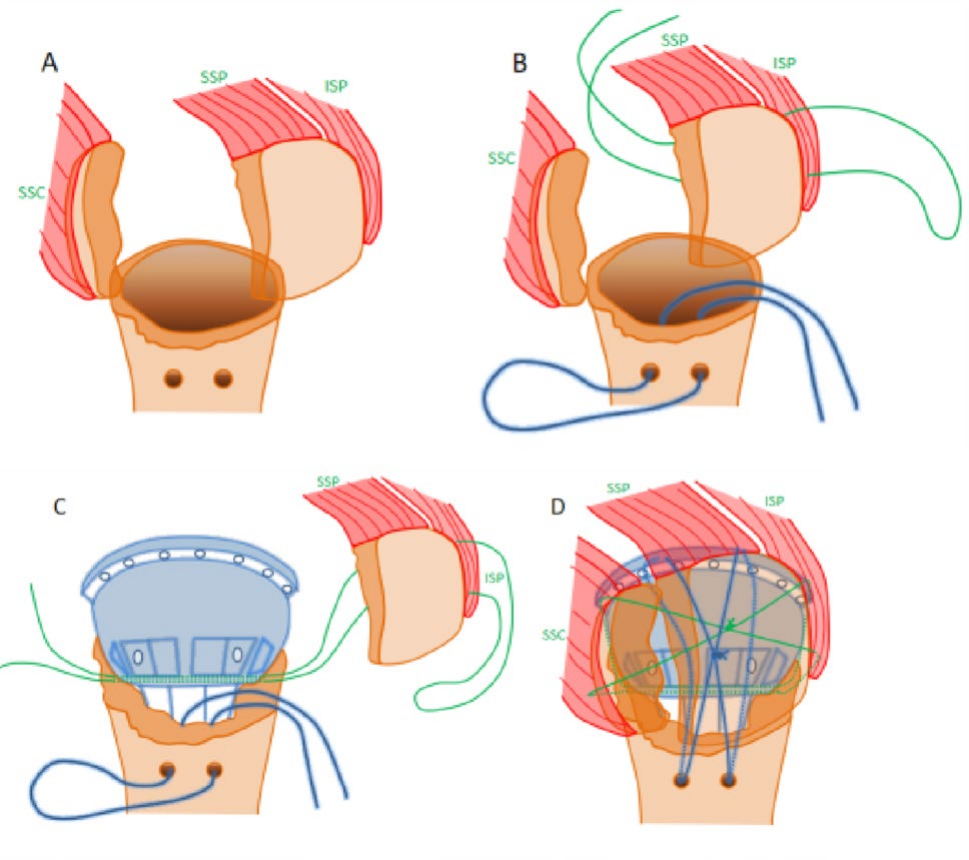
Schematic illustration of the two-suture greater tuberosity refixation technique used in reverse shoulder arthroplasty for proximal humerus fractures.** a** Identification of the greater tuberosity fragments and preservation of the attached rotator cuff tendons,** b** Placement of two high-strength sutures around the greater tuberosity fragment and through the rotator cuff tendon–bone unit,** c** Introduction of the prosthetic epiphysis and anatomical reduction of the greater tuberosity fragment around the fracture-specific component,** d** Final fixation using a horizontal cerclage suture around the prosthetic epiphysis and a vertical tension-band suture passed through drill holes in the humeral shaft to counteract superior migration

A standardized operative procedure was followed, with all procedures performed by one of two surgeons (R.M., and F.H.) using the Global Unite Fracture Reverse System (DePuy Synthes, Warsaw, Indiana, USA). Patients received general anesthesia in combination with an interscalene nerve block and were placed in the beach chair position. The deltopectoral approach was used, involving meticulous dissection and preparation of the greater tuberosity. Biceps tenodesis was performed, and supraspinatus tendons were preserved when present. The GT fragment was identified and refixation was attempted in all included cases using a standardized two-suture construct (Fig. 1a-d). Any rotator cuff tissue remaining attached to the GT (in particular the infraspinatus/teres minor tendon insertion) was preserved and incorporated into the fixation. No routine detachment of cuff tissue from the fragment was performed. In cases with marked comminution or small tuberosity fragments, fixation was performed through the tendon–bone unit to restore continuity to the tuberosity bed and to stabilize the metaphyseal grafted region.

After removal of the humeral head fragment, the glenoid was exposed, and a circular capsular release was performed. The metaglene was implanted and secured using angular stable screws. A detailed description of the technique has been previously published in “*Tuberosity union in patients with proximal humerus fractures treated with reverse shoulder arthroplasty: a technical note and exploratory analysis”* [13]. In brief, GT refixation was performed using a standardized two-suture construct. Autologous cancellous bone graft harvested from the resected humeral head was placed in the metaphyseal region and GT bed prior to refixation.

The GT fragment was reduced anatomically against the fracture-specific epiphysis. The first high-strength suture was placed in a cerclage configuration around the GT and through the prosthetic epiphysis to provide horizontal compression. The second suture was passed vertically through drill holes in the humeral shaft and tied over the GT to create a tension-band effect counteracting superior migration. In cases of comminution or small fragments, the sutures were passed through the attached rotator cuff tendon–bone unit to secure the GT to the grafted metaphyseal bed. Cement interposition in the proximal fixation zone was avoided in cemented stems.

Cemented fixation was performed in fractures involving the proximal diaphysis, where a reliable metaphyseal press-fit could not be achieved. In these cases, approximately 20 mL of standard (non-antibiotic) Palacos PMMA cement (*Heraeus Medical*) was applied distally in the humeral canal to ensure adequate axial and rotational stability. No cement was introduced proximally to preserve the GT fixation zone. Instead, the metaphyseal region and the GT bed were reconstructed using autologous cancellous bone graft, following the same technique applied in uncemented stem implantation. Uncemented stems were used when metaphyseal bone stock permitted stable press-fit fixation without cement augmentation.

Postoperatively, patients were instructed to wear a neutral rotation sling for six weeks. Passive range-of-motion exercises began on the first postoperative day. After six weeks, the sling was discontinued, and active range of motion was allowed, excluding heavy lifting. Muscle-strengthening exercises, sports, and other physical activities were gradually introduced over the following three months.

## Data collection and assessments

Baseline demographic data were collected from the electronic medical records (KISIM; Cistec, Zurich, Switzerland), including age, sex, side of injury, injury on dominant arm, American Society of Anesthesiologists (ASA, I-IV) physical status classification, and body mass index (BMI, kg/m^2^). Additional data on hospitalization were recorded, such as length of stay and time from hospital admission to surgery. Fracture-related characteristics were also documented, including classification and fixation method (cemented vs. uncemented).

As part of the standard of care at our institutions, all patients underwent comprehensive clinical and radiological assessments prior to surgery and at 6 weeks, 12 weeks, 6 months, 12 months, and 24 months postoperatively. Although data were collected at each interval, only the final two-year outcomes are reported in this study, as short-term results with a smaller cohort have been published previously [13]. The current analysis focuses on whether the improvements observed early after surgery were sustained at two-year follow-up. Clinical assessments included measurements of maximum active range of motion (ROM, degrees), Constant-Murley Score (CS), Subjective Shoulder Value (SSV), and American Shoulder and Elbow Surgeons (ASES, 0-100) score. Radiological assessments included GT consolidation, inferior scapular notching (graded according to Nerot-Sirveaux classification), radiolucent lines, humeral stem loosening, DTI, and ossification. All radiographic evaluations were performed by a single musculoskeletal radiologist using predefined criteria. The assessor was not involved in the surgical procedures but was not blinded to clinical data or stem fixation type. Interrater reliability was not assessed.

Greater tuberosity healing was categorized as anatomic healing, malunion, or dislocation [27]. Anatomic healing was defined as the GT tips being located below the tip of the prosthesis. Malunion was recorded when the tuberosity healed but was positioned above the prosthesis tip. Dislocation was defined as the absence of contact between the greater tuberosity and the humeral shaft. This category also captured cases in which the greater tuberosity did not consolidate in continuity with the shaft despite attempted refixation.

### Statistical analysis

The primary endpoint was GT healing at two years. A one-sample proportion power calculation was performed using a two-sided α of 0.05 and 80% power. Based on published GT healing rates after RSA for fractures, a reference proportion of 70% was assumed, and an absolute increase to 85% was considered clinically relevant (delta = 15%). This resulted in a required sample size of 64 patients. To account for potential loss to follow-up, the target sample size was increased by approximately 10% (*n* = 71). The final cohort of 73 patients met this requirement.

Descriptive analyses were conducted for baseline demographics, hospitalization data, surgical details, and clinical/radiological parameters. Categorical variables are reported as absolute and relative frequencies (n, %), whereas continuous variables are summarized as means with standard deviations (SD) or medians with interquartile ranges (IQR), depending on data distribution. Between-group comparisons were conducted using the chi-square test or Fisher’s exact test for categorical data and the independent t-test or Mann-Whitney U test for continuous data, as appropriate. Data normality was assessed visually and using the Shapiro-Wilk test. All statistical tests were two-sided, and a p-value of < 0.05 was considered statistically significant. Analyses were performed using Stata, version 15.0 (StataCorp, College Station, TX, USA).

## Results

Of 94 patients screened for eligibility, 73 met the inclusion criteria and provided informed consent to participate in this study. Of the 21 excluded cases, one involved revision surgery, and the remaining 20 underwent surgery with a different prosthetic system. Patient characteristics and baseline parameters of the fractures are presented in Table 1. According to the Neer classification, 28 (38%) patients had three-part fractures, 20 (28%) had two-part fractures, 18 (24.7%) had four-part fractures, six (8%) had head-split fractures, and one (1.3%) patient presented with a secondary dislocation of the humeral head following initial conservative treatment. Of these surgeries, 19 (26%) were performed with cemented fixation and 54 (74%) with uncemented fixation. No statistically significant differences in baseline parameters were observed between the two fixation groups.

**Table 1:** Baseline demographic and clinical characteristics of the study cohort by fixation group (*n* = 73)

Parameter	Total cohort (*n* = 73)	Cemented (*n* = 19)	Uncemented (*n* = 54)	Intergroup comparison (*p*-value)
Mean (±) age (years)	79 (8)	80 (8)	78.6 (8)	0.467
Gender (female) – no. (%)	65 (89)	18 (94.7)	47 (87.0)	0.671
Side of injury (right) – no. (%)	31 (42)	9 (47.2)	22 (40.7)	0.788
Dominant arm injured – no. (%)	62 (85)	15 (78.9)	47 (87.0)	0.461
Median (IQR) BMI (kg/m^2^)	26.6 (23–30)	26 (24–28)	27 (24–31)	0.204
Mean (±) ASA (I-IV)	2.7 (0.7)	2.6 (0.6)	2.7 (0.7)	0.437
Median (IQR) length of hospital stay (days)	8 (6–10)	9 (6–10)	8 (7–11)	0.681
Median (IQR) time from admission to surgery (days)	3 (2–6)	3 (1–4)	3.5 (2–6)	0.093
Fracture type (Neer) – no. (%)				
Head split fractures	6 (8)	1 (5.3)	5 (9.3)	0.479
Two-Part	20 (28)	8 (42.1)	12 (22.2)	
Three-Part	28 (38)	5 (26.3)	23 (42.6)	
Four-Part	18 (24.7)	5 (26.3)	13 (24.1)	
Other	1 (1.3)	0 (0.0)	1 (1.9)	

*BMI* body mass index,* ASA* American Society of Anesthesiology score,* IQR* interquartile range

## Clinical and radiological results at two-year follow-up

Table 2 summarizes the clinical outcomes according to the fixation types at the two-year follow-up assessments. Patients treated with cemented fixation demonstrated significantly greater forward flexion (*p* = 0.045) and abduction (*p* = 0.02), while all other functional parameters were comparable between the groups.

**Table 2:** Clinical results at two years after surgery with cemented fixation versus uncemented fixation

Clinical parameter	Total cohort (*n* = 73)	Cemented (*n* = 19)	Uncemented (*n* = 54)	Intergroup comparison (*p*-value)
Median range of motion (IQR) - °				
Abduction	140 (120–160)	155 (140–165)	140 (120–160)	0.0447
Flexion	140 (120–160)	155 (140–165)	140 (120–160)	0.02
ER1	30 (20–40)	30 (20–40)	30 (20–40)	0.735
ER2	80 (70–80)	80 (70–80)	80 (70–80)	0.8462
CS score:				
Pain	15 (14–15)	15 (14–15)	15 (15–15)	0.2929
ADL	20 (19–20)	20 (18–20)	20 (19–20)	0.556
Flexion	8 (8–10)	10 (8–10)	8 (8–10)	0.1138
Abduction	8 (8–10)	10 (8–10)	8 (8–10)	0.2558
External rotation	8 (8–8)	8 (8–8)	8 (8–8)	0.5224
Internal rotation	6 (6–8)	8 (6–8)	6 (4–8)	0.375
Strength	9 (8–10)	8 (8–11)	9 (8–10)	0.7964
*Total CS*	76 (73–81)	76 (73–79)	76.5 (72–81)	0.8745
SSV score	90 (80–95)	90 (80–95)	90 (80–95)	0.9795
ASES Score	89.9 (82-93.3)	89.9 (81.6–94.9)	89.9 (82–93)	0.6569

*IQR* interquartile range,* CS* Constant-Murley score,* SSV* Subjective Shoulder Score,* ASES* American Shoulder and Elbow Surgeons,* ER1* External Rotation in Neutral Position,* ER2* External Rotation in 90° of Abduction,* ADL* Activities of Daily Living

Table 3 presents the radiological outcomes. One patient in the overall cohort developed early symptomatic humeral stem loosening requiring revision surgery prior to the scheduled radiographic follow-up and was therefore excluded from the radiologic loosening assessment. Among the remaining patients who completed the two-year radiographic evaluation, one case showed non-progressive radiolucent lines around the humeral shaft without migration, which did not meet the criteria for stem loosening. Consequently, no radiologic loosening was identified in any patient who completed the two-year radiographic follow-up.

**Table 3:** Radiological findings two years after surgery with cemented fixation versus uncemented fixation

Clinical parameter	Total cohort (*n* = 73)	Cemented (*n* = 19)	Uncemented (*n* = 54)	Intergroup comparison (*p*-value)
Healing of greater tuberosity (GT) – no. (%)				
Anatomic	58 (79)	15 (78.9)	43 (79.6)	0.775
Malunion	10 (14)	2 (10.5)	8 (14.8)	
Dislocated	5 (7)	2 (10.5)	3 (5.6)	
Scapular notching – no. (%)				
Grade 0	59 (81)	16 (84.2)	43 (79.6)	0.999
Grade 1	10 (14)	2 (10.5)	8 (14.8)	
Grade 2	3 (4)	1 (5.3)	2 (3.7)	
Grade 3	1 (1)	0 (0)	1 (1.9)	
Heterotopic ossification – no. (%)	5 (7)	3 (15.8)	2 (3.7)	0.107
Radiographically identified stem loosening – no. (%)	0 (0)	0 (0)	0 (0)	–

Scapular notching, classified according to the Nerot–Sirveaux scale, was observed in 14 (19%) patients: 10 (14%) Grade 1, 3 (4%) Grade 2, and 1 (1%) Grade 3. GT dislocation occurred in five (7%) patients: two (10.5%) in the cemented group and three (5.6%) in the uncemented group, with no statistically significant difference (*p* = 0.78).

### Complications

Four (5.5%) patients experienced complications. Two sustained acromion fractures at 6 and 9 months postoperatively, without any preceding trauma. One patient suffered a traumatic coracoid process fracture following a fall. The remaining complication involved one patient in the uncemented group who developed early symptomatic humeral stem loosening due to intraoperative undersizing; this occurred before the scheduled radiographic follow-up and therefore required revision surgery prior to completion of the two-year assessment.

## Discussion

This prospective cohort study evaluated the clinical and radiological outcomes of a simplified tuberosity refixation technique using the Global Unite Fracture Reverse System in patients undergoing reverse shoulder arthroplasty for complex proximal humerus fractures. With a follow-up period of two years, our findings demonstrate high rates of GT healing (93%), low complication rates (5.5%), and favourable functional outcomes. These results are at the upper end of GT healing rates reported in the literature, which range from 37% to 84.6% [13, 19–22, 28]. GT refixation was attempted in all included cases using the standardized two-suture construct. When fragments were small or comminuted, fixation was achieved by incorporating the attached rotator cuff tendon–bone unit and compressing it against the grafted metaphyseal bed rather than relying solely on bony purchase.

In this study, we defined successful healing not only as an anatomically healed GT positioned below the tip of the metaphysis but also included cases where the GT healed slightly above the metaphysis tip. Recent evidence indicates that such minor elevation does not adversely affect function, with outcomes comparable to anatomically positioned healing. Furthermore, the study found that some GTs initially classified as anatomically healed exhibited minor plastic deformation between 6 and 24 months without compromising clinical outcomes, likely reflecting compromised blood supply and normal bone remodelling processes [29].

Healing rates following RSA vary widely across studies. A meta-analysis by Jain et al., which included seven studies encompassing 382 patients, reported an overall average GT healing rate of 68.3% (± 15.9) [6, 8, 15–17, 30–33]. Chun et al. reported a relatively low GT healing rate of 37%, whereas Grubhofer et al. observed a substantially higher GT rate of 84% [15, 31]. Our results, consistent with previous findings using the same technique (90% GT healing at one year) [13], demonstrate reproducible healing at mid-term follow-up. This present study reinforces this positive trend, demonstrating a two-year GT healing rate of 93%. Greater tuberosity healing rates were comparable between cemented (89.5%) and uncemented (94.4%) stems, supporting the technique’s reliability even in osteoporotic bone and without relying on cement for stability.

Our surgical technique emphasizes simplicity, reproducibility, and efficiency. Using only two high-strength sutures, avoiding excessive soft-tissue stripping, and employing a fracture-specific epiphysis collectively create a stable construct that supports biological ingrowth. Essential technical aspects include rigid fixation of the tuberosities against the epiphysis, additional anchorage via shaft drill holes, and liberal use of bone grafting to prevent migration [34, 35]. During cementation, attention is directed toward avoiding contact with the most proximal zone to preserve the integrity of the GT fixation and focus on stabilizing the underlying bone graft [35–37]. The time-efficient nature of the technique is advantageous for elderly patients, for whom prolonged anesthesia and soft-tissue handling carry increased risks. Despite the lack of evidence in the literature indicating fracture-specific stems outperform conventional reverse stems, the open metaphyseal design may improve bone grafting and offer additional fixation points.

Clinical outcomes aligned with existing literature showing satisfactory function after RSA for proximal humerus fractures with both fixation techniques. A meta-analysis by Huang et al., which reviewed 3,150 studies including 14 randomized controlled trials and 791 shoulders with complex proximal humeral fractures, confirmed RSA as the preferred treatment modality in terms of Constant-Murley score and ROM (forward flexion and outreach) [38]. Similarly, a meta-analysis by Rossi et al. comparing cemented and uncemented RSA reported similar outcomes in ROM, functional scores, and tuberosity healing [18], findings that align with our results. However, Rossi et al. also found a significantly higher complication rate in the uncemented group (9.7%) compared to the cemented cohort (5.5%) [18], a difference that was not observed in our cohort. Kramer et al. noted higher proximal bone resorption with uncemented stems, though without functional consequences [26]. In our cohort, despite 70% of patients showing poor bone quality (DTI < 1.4), fixation method did not influence clinical outcomes or complication risk, supporting the use of uncemented stems in osteoporotic bone when stable fixation is achieved [27].

This study has limitations. First, GT healing was assessed using plain radiographs rather than computed tomography (CT), which may reduce diagnostic precision in detecting subtle displacement or nonunion. However, radiographs are commonly used in clinical follow-up after reverse shoulder arthroplasty for fractures and allow standardized serial assessments with lower radiation exposure. Second, radiographic assessments were performed by a single non-blinded observer, which may introduce observer bias and limits the ability to assess interrater reliability. Third, although two-year follow-up provides valuable mid-term data, long-term changes such as tuberosity resorption, late humeral stem loosening, or functional decline may become evident only with extended follow-up of at least five years. Fourth, minimally clinically important difference (MCID) analyses were not performed because (a) MCID is based on within-patient change and preoperative functional values in the acute fracture setting do not represent true baseline shoulder function, and (b) the present report focuses on final two-year outcomes. Fifth, although the sample size calculation confirmed adequate power for the primary endpoint of GT healing, the study was not powered for comparisons between cemented and uncemented stems, and these analyses should therefore be interpreted as exploratory. Likewise, group allocation was not randomized, introducing potential selection bias, even if baseline characteristics were similar. Further research is warranted to clarify the role of cementation specifically within this simplified refixation framework. Finally, the lesser tuberosity was not evaluated, potentially overlooking its contribution to shoulder function and stability.

## Conclusion

This study demonstrates that reverse shoulder arthroplasty, combined with the evaluated tuberosity refixation technique, is a safe and effective procedure for complex proximal humerus fractures in elderly patients. The technique achieved a 93% GT healing rate at two years, with minimal complications and consistently favorable outcomes. Results were comparable regardless of bone quality or fixation method, highlighting the reproducibility and clinical practicality of the technique. The findings further support the use of uncemented stems in osteoporotic patients when stable fixation and reliable tuberosity refixation can be achieved. 

## Data Availability

No datasets were generated or analysed during the current study.
